# Notes of Surgical Cases—Removal of the Left Half of the Inferior Maxilla

**Published:** 1863-01

**Authors:** S. Barrett

**Affiliations:** Le Roy, Genesee Co.


ART. III.—*Notes of Surgical Cases—Removal of the left half of the Inferior Maxilla. By* S. BARRETT, M. D., *of Le Roy, Genesee Co.*

Mrs. E. D. C., of this place, aged 57, has suffered since nineteen years of age from disease of the left side of the superior maxillary bone. At the commencement of the disease, and for many years after, she was a resident of Boston, Mass., and under the care of Dr. John Ware; but subsequently removed to this place. The disease commenced near the centre of the left side of the jaw; the pain came on in paroxysms of great severity, and could only be relieved by large doses of anodynes. Some few years after the local pain commenced the bone began to enlarge at the point where the severe pain centered, producing a slight degree of deformity of that side of the face. The pain gradually extended over the side of the face and head; the cutaneous nerves became exceedingly sensitive; so much so that the least current of cool air gave her the most excruciating pain, and several times during the progress of the disease she was reduced very low, her life being quite despaired of; but she would gradually rally, and enjoyed some intervals of more tolerable health. Soon after the bone began to enlarge she found some difficulty in opening the mouth, and had the teeth extracted on that side. The disease gradually extended to the joint, and for more than twenty years there had been complete anchylosis. In June, 1860, she came under my care; she had then been unable to leave the house but very little for the previous year. There was great nervous prostration and general debility; she had become emaciated and worn out with pain, and was mostly confined to her bed. By the use of tonics and nervines, with as good diet as the stomach would bear, she gradually rallied; and on the 18th of July following, assisted by Drs. Chamberlain and Smith of this place, and Dr. Williams of Byron I removed the left half of the jaw.—After bringing her fully under the influence of chloroform, I made an incision from the angle of the jaw to the centre of the chin; then pierced the lip just below its edge so as to avoid the labial artery, carried the incision down to meet the other below the chin; dissected the flaps up from the bone, and tied the facial artery as I divided it; then sawed the bone at its symphasis with a small Hayes saw. I then carried the incision up to the joint; separated the periostium around the neck of the bone; found it enlarged and perfectly ossified through the joint. The zygomatic process was enlarged, and formed with the jaw one solid bone. I placed the handle of a scalpel so as to protect the ear, and with the small Hayes saw. at the junction of the two bones, sawed into the joint so as to enable me by a little effort to break it out. It severed the internal maxillary artery, which seemed to be enlarged, and bled pretty feeely, From its deep position it gave us some trouble in securing it, but by seizing it with the forceps and then passing a small tenaculum through it beyond, we succeeded in ligating it. The flaps were brought together and secured by a few interrupted sutures and adhesive straps. The wound healed kindly, except some delay occasioned by a pretty smart attack of erysipelas, passing over the entire face and head. Her health has been gradually improving up to this time. There is but little deformity from the operation, and she can use the other side of the jaw to masticate her food tolerably well. The entire bone removed was in an ebernated or ivory condition, and a longitudinal section showed the cancellated structure to be entirely obliterated, which explained the cause of so much suffering, as the dental nerve during the progress of the disease must have suffered continual and increasing pressure. The temporal and malar bones were both involved to some extent in the disease, but little pain however has been felt up to this time in them, and no indications of the disease progressing in other parts.

